# Impact of Different Illumination Levels on Ocular Parameters in Healthy Adults During Book-Reading: An Experimental Study

**DOI:** 10.22599/bioj.529

**Published:** 2026-04-01

**Authors:** Vishal Biswas, Roshni Majumder, Supriya Awasthi, Rahul Singh

**Affiliations:** 1 Department of Optometry, School of Allied Health Sciences, Noida International University, India; 2 School of Allied Health Sciences, Noida International University, India; 3 Department of Ophthalmology, Noida International Institute of Medical Sciences, Noida International University, Uttar Pradesh, India

**Keywords:** Room illumination, Binocular vision, Accommodation, Convergence

## Abstract

**Aim:**

To determine the role of different levels of illumination on various binocular vision parameters.

**Settings and design:**

An experimental study was conducted from October 2024–June 2025.

**Methods:**

Thirty-four participants aged between 17 to 25 years with a mean age of 19.7 ± 2.4 years were included. Under different lighting conditions, i.e., low illumination (50 lux), medium illumination (100 lux), and high illumination (150 lux), the participants were instructed to perform reading tasks. The vergence and accommodative parameters for near were evaluated.

**Results:**

Observed changes in binocular vision parameters were associated with varying illumination levels. At 50 lux, the binocular accommodative facility (BAF) was 6.97 ± 0.86 cpm; at 150 lux, it was 10.55 ± 1.04 cpm. Higher illumination significantly enhanced vergence facility (VF), near point of accommodation (NPA), and near point of convergence (NPC). Brighter illumination enhanced both positive and negative fusional vergence (PFV and NFV) values, while MEM retinoscopy demonstrated decrease of accommodative lag.

**Conclusions:**

Binocular vision parameters were higher significantly with high illumination which can enhance visual performance. Proper lighting is essential for eye exams, digital device use, and workspace design.

## Introduction

Illumination is an important aspect that impacts the productivity of an individual at the workplace (Tupala, 2025). The levels and type of illumination is dependent upon the nature of work and environment of the workplace. Various studies have been conducted recommend the amount of appropriate illumination necessary for improved productivity at workplace. Numerous studies have stated different lighting requirements for different types of works such as 300–500 lux for general office working (Rasouli Kahaki *et al*., 2022). In laboratory-based studies evaluating mental workload, an illumination level of approximately 750 lux is recommended (Bao *et al*., 2021).

According to the 2011 National Electric Code of Indian standards, Standard Code of Practice for Industrial Lighting recommends 300–400 lux for reading and writing, 300–700 lux for reading, and 200–500 lux for tasks like reading (Ram and Bhardwaj, 2018). Studies investigating the effect of illumination on binocular function have reported mixed outcomes. Chen *et al*. (2017) found that using a 3D target under brighter lighting resulted in greater accommodative, than convergence, response, while Bai-Chuan Jiang *et al*. (1991) reported no difference under average illumination. According to Okada *et al*. (2013), when environmental illumination was reduced to low levels (e.g., below approximately 100 lux), the accommodative and convergence demand increased, whereas under brighter lighting conditions (around 500 lux and above), individuals required less accommodative and convergence effort.

Similarly, Azam *et al*. (2023) observed higher PFV in lower lighting, in contrast to Majumder and Zafirah Zaimi (2017) who reported no clinically significant effect on accommodation. It has been shown that the right illumination may help mitigate negative impacts, improve academic achievement, and resolve problems. Behaviour, academic achievement, and professional success may all be strongly impacted by brightness. It has been shown that brighter lighting lowers stressful workloads and increases productivity (Abramson *et al*., 2007).

Previous studies examining the effects of illumination on ocular accommodation and convergence have reported inconsistent findings. There is no consensus on whether changes in lighting levels significantly influence accommodative or vergence parameters. Therefore, the present study aimed to evaluate key binocular vision measures under varying illumination conditions to determine the potential impact of lighting on visual function in healthy adults.

## Methods

An experimental study was conducted from October 2024–June 2025 using within-subject design for evaluating the effects of different levels of illumination on ocular parameters. Ethical review was granted from the institutional ethics committee of Noida International Institute of Medical Sciences, Greater Noida, India (NIIMS/IEC/OCT24/R-34) and the research protocol was registered with the Clinical Trial registry India under identifier dated 24.3.25 (CTRI/2025/03/083170). Participation was entirely voluntary in this study. All the participants received a detailed explanation regarding the purpose of the study, procedure, potential risks, and benefits. Written informed consents were obtained from each participant before the commencement of the study. The participants were informed about their rights to withdraw from the study at any time without providing any reason.

The study followed similar methods, as per the study by Azam *et al*. (2023), though the screening process of the participants included distance visual acuity assessment with Log Mar chart for distance and near. The visual acuity criteria were set to be Best Corrected Visual Acuity (BCVA) of at least 0.1 for distance and N6 for near vision, followed by anterior and posterior segment evaluation with slit-lamp biomicroscope and 90 D, to rule out individuals with any existence of ocular pathology. A complete binocular vision evaluation was performed to rule out individuals with the sort of anomalies related to binocular vision. And individuals with any systemic illness were excluded.

### Sample size calculation

Sample size calculation was done using Fisher & Law: (Jung, 2014)

n = Sample size, Z = Standard normal variant = 1.96 (If we consider 95% confidence interval then Z score is 1.96.) P = Expected proportion in population based on pilot studies (0.021) d = Absolute error or precision of 5% (0.05)

Sample size calculation = ((1.96)²* 0.020* (1–0.020))/(0.05)² = 30.11~31

Considering 10% drop out, the calculated sample size was 34.

### Illumination used

The study was carried out in accordance with established ergonomic lighting guidelines recommended for workplace illumination standards for visual tasks (ISO, 2025). Fifteen-watt fluorescent bulbs mounted on the ceiling as a part of the lighting setup. Separate switches were placed for each bulb; it allowed the examiner to control the level of illumination as per the experimental requirements.

A lux meter (Brand: Sigma Instruments measurement range: 1–50,000 lux across three selectable ranges) was used to measure the level of illumination throughout the experiment. The measurements were taken at eye level of each participant to maintain uniformity regarding the illumination level measurement.

We conducted our study in three phases; phase 1 (low illumination), a single ceiling-mounted bulb provided illumination of 50 lux; phase 2 (medium illumination) two ceiling-mounted bulbs yielding illumination of 100 lux; and phase 3 (high illumination) involved three ceiling bulbs producing illumination of 150 lux. The sequence of these illumination levels was counterbalanced across the participants. Although these values are lower than standard recommendations for near tasks (typically 300–500 lux according to ISO 8995-1:2002), they were purposefully chosen to simulate suboptimal lighting conditions that are commonly encountered in real-world settings, as referenced in a previous study on visual performance under low illumination (Azam *et al*., 2023).

### Outcome measures

To assess the amount of deviation, the cover test was performed using a horizontal prism bar with incremental prism powers ranging from 1 prism dioptre to 40 prism dioptre. Broad H test was used to evaluate the ocular motility.

#### Vergence parameters

The Royal Air Force (RAF) ruler was used to measure NPC, where a line target was concentrated on the retina. By averaging three readings, the findings dependability was assessed (Biswas and Majumder, 2024).

PFV and NFV was measured at a near distance of 33 cm using a horizontal prism bar. The near (for measuring near fusional vergences) parts of the near log Mar chart, a set of 20/30 letters, were employed, grouped in a column. The positive and negative fusional break points and recovery points were evaluated using base-out (BO) and base-in (BI) prisms (Biswas and Majumder, 2024).

Vergence facility (VF) refers to the responsiveness and efficiency of the fusional vergence system in adjusting to varying vergence demands over time. It is quantified in cycles per minute (cpm), which is the number of times in a minute that an individual can fuse a picture with alternating base-in (BI) and base-out (BO) prism stimuli. In this study, participants read N6-sized text while having their VF measured at 40cm using prism flippers equipped with 12 prism dioptres (PD) BO and 3 PD BI (Biswas and Majumder, 2024).

#### Accommodative parameters

The NPA was assessed using the push-up test with both eyes open and monocularly (right and left eye) (Mark Rosenfield, 2016). For binocular viewing, the accommodative facility was measured and represented in cycles per minute, ±2.00 D accommodative flipper was used for this assessment. This assessment was done binocularly (Chikuse *et al*., 2022). The MEM was used to evaluate the lead and lag of accommodation utilizing test targets of short, three-letter phrases in N8 font size. A typical lag was thought to be in the range of +0.25 to +0.75D (TASSINARI, 2002).

## Experiment Setup

As a part of the experimental procedure, participants were instructed to complete a standardized book-reading task under variable lighting conditions. The reading material was ‘The Little Prince’ by Antoine de Saint-Exupéry, this narrative material was selected due to its simple language and broad appeal. The Flesch-Kincaid grade level ensured minimal cognitive level requirement for adult readers with a score of 5.5. The size of the reading material was 12-point, Times New Roman font. To reduce the glare and increase the readability the material was printed in black ink on a matte white background. To minimize potential glare, additional lighting sources were omitted (Solnyshkina *et al*., 2017).

The participants were comfortably seated at a constant reading distance of 40 cm, with the reading material positioned on a table ergonomically. For a duration of 30 minutes under each illumination the participants were instructed to read the material. The reading distance was monitored every 10 minutes by the primary investigator to maintain consistency.

Different excerpts of the books in each lighting phase were read by the participants to avoid content repetition and maintaining engagement. Each participant read different excerpts of the book in each lighting phase to avoid content repetition and maintain engagement.

For each illumination, the 30 minute reading task was followed immediately by the visual tests (vergence parameters first followed by accommodative parameters). Consistency in visual demand and environmental variables was maintained by performing the evaluations under the same light level as the reading task. Through this process, it was made sure that the measured parameters accurately represented the impact of the lighting environment under test.

To reduce the risk of observer bias, the examiner who assessed all the parameters was masked to the illumination conditions. To reduce the possibility of biasness, the principal investigator masked the examiner who assessed pre and post binocular vision parameters of the study participants.

### Statistical analysis

IBM SPSS Statistics for Windows, version 29.0 (IBM Corp., Armonk, NY, USA) was used for inferential and descriptive statistics. Shapiro-wilk test was used for checking the normality of the data set. Since the data did not follow a normal distribution, Friedman’s test was used across all three illuminations to compare the ocular parameters. The Wilcoxon signed rank test was employed to compare two distinct illumination levels (50 and 100, 100 and 150, and 50 and 150 lux).

## Results

Over a period of eight months, 34 participants completed the study. The mean age of the subjects was 19.7 ± 2.4 years. Most of the participants were male n = 21 (61.7%).

**Figure 1:**
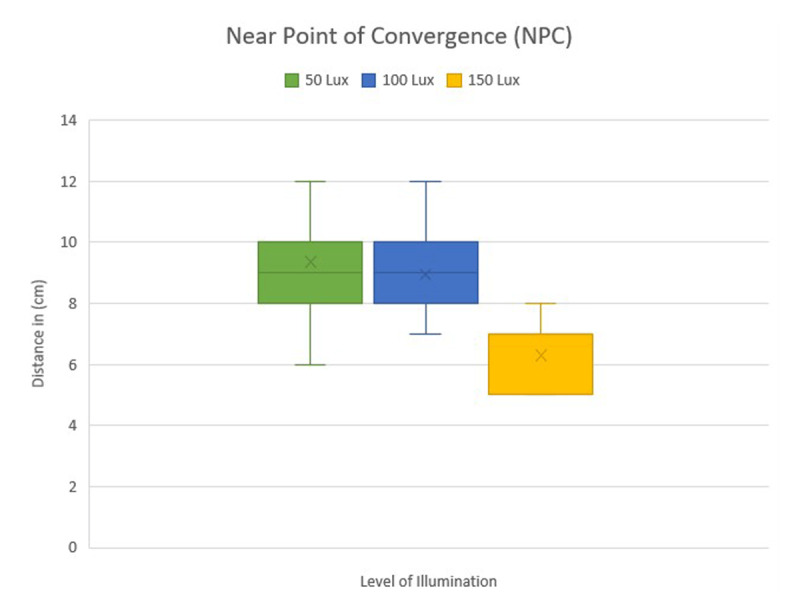
Comparison of NPC among three different illumination levels.

VF increased from 8.26 ± 1.08 cycles per minute at 50 lux to 10.2 ± 0.94 at 150 lux. Additionally, there was a notable improvement in NPC, as the mean value decreased from 9.35 ± 1.47 cm at 50 lux to 6.29 ± 0.97 cm at 150 lux. The measurements of the NPC in different levels of illumination are shown in (Figure 1).

**Figure 2:**
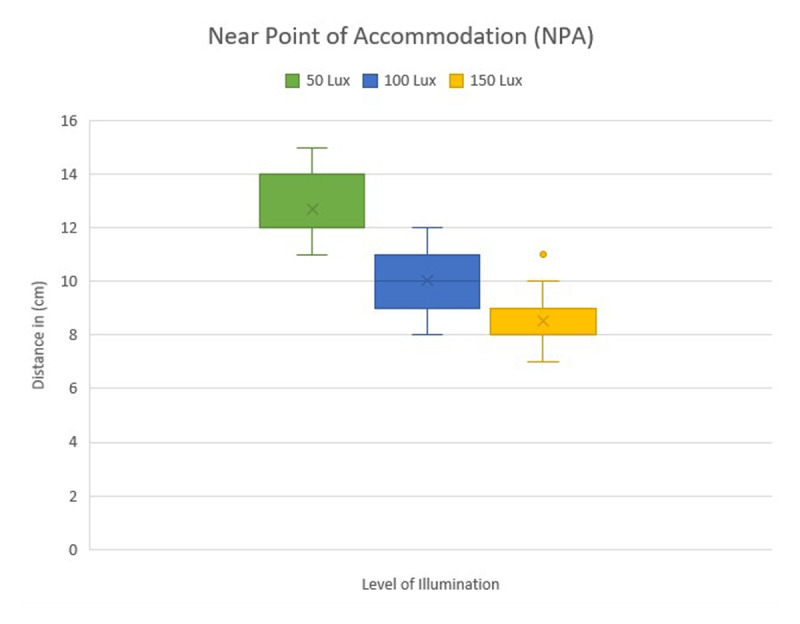
Comparison of NPA among three different illumination levels.

With increase in illumination, PFV and NFV values at near exhibited numerical increases in BLR, BR, and REC (e.g., PFV-BLR: 10.28 ± 5.39 at 50 lux vs. 13.28 ± 8.38 at 150 lux), all of which were p > 0.001. More light gradually increased the mean values of the BAF, which increased from 6.97 ± 0.86 cycles per minute at 50 lux to 8.76 ± 1.07 at 100 lux and 10.55 ± 1.04 at 150 lux. NPA decreased from 12.7 ± 1.16 cm at 50 lux to 8.52 ± 1.02 cm at 150 lux, indicating enhanced accommodative function. The measurements of the NPA in different levels of illumination is shown in (Figure 2).

MEM retinoscopy showed that both eyes’ accommodative lag decreased as light increased. The left eye (LE) showed a similar pattern, with the right eye (RE) showing a decrease from 0.74 ± 0.25 D at 50 lux to 0.39 ± 0.16 D at 150 lux.

Statistically significant differences were observed across all parameters with changing illumination levels (p < 0.001) (Table 1).

A pairwise comparison utilizing the Wilcoxon-signed rank t-test revealed no significant difference between 50 and 100 lux for NPC (p = 0.13). However, substantial improvements were detected between 100 lux and 150 lux (p < 0.001) and between 50 lux and 150 lux (p < 0.001). The mean NPC decreased from 9.35 ± 1.47cm at 50 lux to 6.29 ± 0.97 cm at 150 lux, demonstrating improved convergence under higher light. NPA revealed statistically significant improvements across all pairwise comparisons (p < 0.001). The mean NPA decreased from 12.7 ± 1.16 cm at 50 lux to 8.52 ± 1.02 cm at 150 lux, indicating improved accommodating capacity in high illumination conditions. Similarly, BAF increased considerably across all lighting levels (p < 0.001), increasing from 6.97 ± 0.86 cycles per minute at 50 lux to 10.55 ± 1.04 at 150 lux. The VF showed considerable improvement across all pairwise comparisons (p < 0.001), with the mean VF increasing from 8.26 ± 1.08 cycles per minute at 50 lux to 10.2 ± 0.94 at 150 lux (Table 2).

## Discussion

This study examined the effects of different illumination levels on key binocular vision parameters in healthy adults. The studied accommodative and vergence parameters improved noticeably with increased illumination level. For example, NPC improved from 9.3 ± 1.4 cm at 50 lux to 6.2 ± 0.9 cm at 150 lux, whereas mean binocular accommodative facility rose from 6.9 ± 0.8 cpm at 50 lux to 10.5 ± 1 cpm at 150 lux.

These changes suggest that higher illumination levels enhance accommodative response and improve vergence stability. Unlike earlier studies that broadly addressed workplace lighting for productivity (Bellia *et al*., 2011; Ram and Bhardwaj, 2018; Shen *et al*., 2009), our results provide quantitative evidence of how specific binocular vision functions respond to varying illumination conditions during a continuous 30 minute reading task. We found that increased light levels greatly improved accommodative responses, which is in line with previous studies (Chen *et al*., 2017; Okada *et al*., 2013). As light intensity increased, the NPA distance decreased, indicating an improvement in accommodative ability in brighter environments. This supports the idea that higher illumination reduces accommodative effort, likely due to pupil constriction and an increased depth of focus (RIPPS *et al*., 1962).

The BAF also shown a significant improvement with high illumination levels, indicating that brighter surroundings could facilitate dynamic accommodative adjustments. These findings corroborate Jiang *et al*.’s report that accommodative effort decreases under high luminance conditions (Bai-Chuan Jiang *et al*., 1991).

Better convergence capacity under high illumination settings was demonstrated by our findings, which also showed a considerable improvement in NPC with high illumination show that greater light levels improve convergence demand, which is consistent with this tendency (Okada *et al*., 2013). Notably, our work found a continuous improvement in both PFV and NFV at near, with increasing illumination. Similar results were found by (Azam *et al*., 2023), showed enhanced PFV under higher illumination levels. It is suggesting that adequate lighting may enhance the efficiency of fusional vergence mechanisms at nearby.

Our findings demonstrated a statistically significant increase in accommodative parameters under higher illumination, which contrasts with a previous study that found no discernible influence of illumination on accommodative parameters. This gap might be explained by methodological discrepancies. While Majumder and Zafirah Zaimi (2017) evaluated monocular amplitude of accommodation using a visual display unit (VDU) at two higher light levels (150 and 300 lux) with brief exposure, our study employed sustained binocular near-vision reading of printed text for 30 minutes under three illumination levels (50, 100, and 150 lux). Extended proximity to printed text in our research design may have increased tiredness and accommodative demand, and variations in visual target type, exposure duration, and illumination range likely contributed to the inconsistent outcomes.

Our findings further illustrate that accommodative lag, as determined by MEM retinoscopy, steadily decreased under brighter illumination, indicating improved accommodative accuracy in well-lit settings. This lends greater support to the idea that lighting helps lessen eye strain when performing near work. Although the values are within the normal range of MEM i.e., +0.25 to +0.75 dioptres (TASSINARI, 2002).

Our research emphasizes how crucial it is to take illumination into account when diagnosing or treating binocular vision abnormalities. Improving lighting may be a straightforward yet efficient way to enhance accommodative and vergence function in clinical and occupational settings, especially while doing jobs that call for prolonged near work at least a minimum lux of 150 is suggested.

### Limitations and future directions

This study only included healthy young individuals in controlled settings, even though the current findings offer strong evidence of the impact of light on binocular vision parameters. The subjects’ limited age range and health state limits the results’ applicability to other populations. To confirm that these findings are generalizable, future studies should include participants of various ages, those with binocular vision impairments, and a variety of task settings. Furthermore, examining subjective symptoms in addition to objective measurements could provide a more thorough comprehension of lighting impacts. Future research could also examine the persistence, diminution, or adaptation of these effects with extended durations or repeated exposure in real-world settings, as the study only evaluated short-term effects (immediately following a 30 minute reading task), leaving it unclear whether these improvements are sustained over longer periods of time or subject to adaptation.

Lastly, the results’ generalizability to other age groups and a more balanced gender distribution is limited by the sample’s homogeneity, which is mostly composed of young adult men.

## Conclusion

This study demonstrates that different levels of illumination have a statistically significant effect on binocular vision characteristics in healthy adults.

Higher illumination was associated with notable improvements, including an average increase of 4.1 cm in NPA, 3.5 cpm in binocular accommodative facility (BAF), and 3.1 cm gain in NPC, along with a 1.9 cpm gain in vergence facility (VF).

These findings indicate that illumination levels up to 150 lux improves visual performance and decreases accommodative and vergence efforts during near activities. Higher level of illumination, which are normal in real-world situations, were not evaluated in our study which could have provided additional advantages because this is below international ergonomic standards for near work (usually 300–500 lux).

Lighting should be considered an important environmental factor when assessing visual performance during sustained near tasks.

## Appendices

**Table 1:** Shows comparison of ocular parameters in different levels of illumination (Friedman test).

PARAMETERS		ILLUMINATION LEVEL									P VALUE

		50 LUX			100 LUX			150 LUX			
		
		MEAN	SD	RANGE	MEAN	SD	RANGE	MEAN	SD	RANGE	

BAF (Cycles per minute)		6.97	0.86	5 to 8	8.76	1.07	7 to 11	10.55	1.04	9 to 11	<0.001

NPA (cm)		12.7	1.16	11 to 15	10.02	1.16	8 to 12	8.52	1.02	7 to 11	<0.001

MEM (Dioptre)	RE	0.74	0.25	0.25 to 1.25	0.5	0.15	0.25 to 0.75	0.39	0.16	0.25 to 0.75	<0.001

	LE	0.72	0.23	0.25 to 1.00	0.51	0.13	0.25 to 0.75	0.38	0.16	0.25 to 0.75	<0.001

VF (Cycles per minute)		8.26	1.08	6 to 10	9.23	0.6	8 to 10	10.2	0.94	9 to 12	<0.001

NPC (cm)		9.35	1.47	6 to 12	8.94	1.01	7 to 12	6.29	0.97	5 to 8	<0.001

PFV-Near (Prism-Dioptre)	BLR	10.17	2.8	5.39 to 15.84	11.97	2.26	7.84 to 16.96	13.88	2.38	8.7 to 18.16	>0.001

	BR	13.21	2.42	7.93 to17.67	15.61	2.1	11.7 to 19.47	16.24	2.04	12.17 to 20.87	<0.001

	REC	10.34	2.34	5.11 to 17.26	11.76	2.44	5.92 to 17.79	12.8	2.32	8.3 to 18.51	<0.001

NFV-Near (Prism-Dioptre)	BLR	7.78	1.75	4.68 to 11.31	9.3	2.37	4.5 to 14.43	10.63	1.82	6.55 to 14.62	<0.001

	BR	9.54	1.65	4.69 to 13.34	11.86	2.65	5.04 to 17.07	13.4	2.91	7.53 to 17.99	<0.001

	REC	6.89	2.38	3.08 to 13.31	8.71	1.78	4.96 to 12.86	9.93	2.49	4.21 to 14.10	>0.001

**Table 2:** Shows comparison of selected parameters in different levels of illumination (Wilcoxon signed rank test).

ILLUMINATION LEVELS	NPC (cm)			NPA (cm)			BAF (CYCLES PER MINUTE)			VF (CYCLES PER MINUTE)		
			
	MEAN	SD	P VALUE	MEAN	SD	P VALUE	MEAN	SD	P VALUE	MEAN	SD	P VALUE

50	9.35	1.47	0.13	12.7	1.16	<0.001	6.97	0.86	>0.001	8.26	1.08	<0.001
			
100	8.94	1.01		10.02	1.16		8.76	1.07		9.23	0.6	

100	8.94	1.01	<0.001	10.02	1.16	<0.001	8.76	1.07	>0.001	9.23	0.6	<0.001
			
150	6.29	0.97		8.52	1.02		10.55	1.04		10.2	0.94	

50	9.35	1.47	<0.001	12.7	1.16	<0.001	6.97	0.86	>0.001	8.26	1.08	<0.001
			
150	6.29	0.97		8.52	1.02		10.55	1.04		10.2	0.94	
